# Synthesis, Characterisation, and Biological Assessment of Chromium Oxide Nanoparticles Coated with Chia Seed Mucilage Extract

**DOI:** 10.3390/pharmaceutics18010049

**Published:** 2025-12-30

**Authors:** Sara Lukač, Nina Tomić, Zoran Stojanović, Vladimir Rajić, Nenad Filipović, Maja Jović, Magdalena Stevanović

**Affiliations:** 1 Group for Biomedical Engineering and Nanobiotechnology, Institute of Technical Sciences of SASA, Kneza Mihaila 35/IV, 11000 Belgrade, Serbia; 2 Department of Atomic Physics, Vinča Institute of Nuclear Sciences, University of Belgrade, Mike Petrovića Alasa 12-14, 11000 Belgrade, Serbia

**Keywords:** chromium oxide nanoparticles, chia mucilage, surface functionalization, physicochemical characterisation, antioxidant properties, biocompatibility

## Abstract

**Background/Objectives**: Chromium (III) oxide nanoparticles possess unique chemical properties, making them increasingly valuable in pharmaceutical applications, which had been neglected until the last few years. However, their use requires stable dispersion and surface functionalization to ensure their biocompatibility. This study aimed to synthesise, characterise, and determine the biocompatibility and antioxidant properties of chromium oxide nanoparticles coated with a natural, plant-derived stabilising agent: chia seed mucilage extract. **Methods**: The synthesised nanoparticles were characterised using X-ray diffraction (XRD), scanning electron microscopy (SEM), energy-dispersive X-ray spectroscopy (EDS), Fourier-transform infrared (FTIR) spectroscopy, and laser diffraction scattering particle size analysis (LD-PSA). Biological and biochemical assessments were conducted by the DPPH and FRAP assays to quantify antioxidant scavenging abilities and the *Artemia salina* lethality test for preliminary biocompatibility evaluation. **Results**: XRD, FTIR, and EDS confirmed the successful synthesis of pure chromium oxide NPs (CrNPs) and their effective coating by the chia mucilage (CM) extract. SEM analysis determined that a 4:1 mass ratio (CrNPs to CM) produced the most consistent morphology and narrowest size distribution, yielding spherical particles approximately 50 nm in diameter. LD-PSA confirmed the coating and identified a hydrodynamic diameter of approximately 0.110 µm. Biological and biochemical assays showed high antioxidant activity, with over 80% free radical scavenging at concentrations of 250 μg/mL and 50 μg/mL. Furthermore, the biocompatibility assessment showed survival rates above 90% across all tested concentrations. **Conclusions**: The findings confirm that chia seed mucilage extract can serve as an effective, biocompatible coating agent for chromium (III) oxide nanoparticles. The resulting functionalized particles exhibit exquisite biocompatibility and significant antioxidant potential, supporting their further development for pharmaceutical use.

## 1. Introduction

Deficiency of the essential trace element chromium has been associated with impaired glucose tolerance, reduced insulin sensitivity, and disruptions in lipid metabolism, physiological disturbances that contribute to the global rise in metabolic disorders such as prediabetes and type 2 diabetes [1,2]. As these conditions continue to increase worldwide and are now recognised as a major public health challenge due to their frequency and severe complications, there is growing interest in identifying micronutrients and molecular pathways that may help counteract metabolic dysregulation. Within this context, trivalent chromium (Cr(III)) has attracted increasing scientific attention [3,4].

Although chromium compounds were historically overlooked in biomedical research, largely due to the well-known toxicity of hexavalent chromium and the resulting misconception that all chromium species pose similar risks, extensive evidence now shows that Cr(III) exhibits far lower toxicity and plays a physiological role in glucose and lipid regulation [5,6]. Recent studies indicate that Cr(III) may potentiate insulin action at the molecular level, particularly in individuals with insulin resistance [7,8]. Chromium (III) is believed to form part of a low-molecular-weight oligopeptide, chromodulin, which interacts with the insulin receptor and enhances its tyrosine kinase activity, thereby improving cellular glucose uptake [9].

Among various trivalent chromium compounds, chromium (III) oxide (Cr_2_O_3_) stands out due to its high chemical stability and the possibility of achieving enhanced biological activity when formulated as nanoparticles [10,11]. Nanostructured Cr_2_O_3_ has demonstrated antibacterial, antioxidant, and anticancer properties, highlighting its potential as a therapeutic agent or as a component of advanced drug delivery systems [10]. Its exceptional chemical durability and biocompatibility have also supported its use in medical devices, including corrosion-resistant coatings for orthopaedic and dental implants and in sensing platforms for pharmaceutical analysis [12].

The unique advantages of metal oxide nanoparticles over their bulk counterparts are, most notably, their dramatically increased surface area, reactivity, and ability to interact with biological systems at the cellular and molecular levels, further justifying interest in chromium (III) oxide nanoparticles for pharmaceutical applications [13,14]. However, to address limitations such as potential agglomeration and to enhance biocompatibility, surface modification or coating is typically required [15,16]. There are various ways of approaching the surface modification of nanoparticles, whether the coating agent is inorganic, lipid, polymeric, or else. While inorganic coatings, specifically silica and carbon-based materials, demonstrate high efficacy in applications involving electronics or optics due to their robustness, their application for biomedical research is often discouraged. This hesitation stems from concerns regarding the potential cytotoxicity and limited biodegradability of these inorganic shells in biological systems [17]. Lipid-based nanocarriers, such as liposomes and solid lipid nanoparticles (SLNs), offer exceptional biocompatibility and biomimetic properties due to their structural similarity to biological membranes. However, their practical application is often limited by complex manufacturing protocols, which are challenging to scale while maintaining batch-to-batch consistency. Furthermore, these systems frequently exhibit thermodynamic instability, leading to premature cargo leakage and drug expulsion [18]. Coating nanoparticles with plant-derived natural polymers enhances their biocompatibility and reduces the risk of premature drug leakage. These biopolymers provide a robust, non-immunogenic shell that mimics the biological environment, facilitating better cellular uptake while maintaining a stable matrix that prevents the burst release effects commonly observed in less stable nanocarriers [19].

Therefore, plant-derived coating agents have emerged as particularly attractive candidates owing to their low toxicity, sustainability, excellent biocompatibility, and intrinsic bioactive properties [20].

Chia (*Salvia hispanica* L.) seed mucilage is one such natural biopolymer, valued for its high water-holding capacity, gel-forming ability, abundance, and low environmental impact [21]. When purified, chia mucilage, composed primarily of complex polysaccharides such as arabinose and xylose [22,23], offers more consistent composition and more stable functional properties than whole-seed extracts, which contain lipids, proteins, and other nutrients that may introduce variability or promote unwanted interactions [24]. Removal of the lipid fraction, particularly omega-3 fatty acids susceptible to oxidation, results in a coating material with improved stability and shelf life [25,26].

Considering the complementary advantages of chromium (III) oxide and purified chia seed mucilage, the main idea of this work was to design and evaluate innovative Cr_2_O_3_ nanoparticles, coated with chia mucilage (CrNPs-CM). The required tasks carried out and reported in this paper were: (i) synthesis of stable chromium nanoparticles CrNPs, (ii) coating of CrNPs with the chia mucilage, (iii) characterisation of the newly synthesised nanoparticles by X-ray diffraction (XRD), scanning electron microscopy (SEM), energy dispersive X-ray spectroscopy (EDS), Fourier transform infrared (FTIR) spectroscopy, laser diffraction particle size analyzer (LD-PSA), (iv) determining the antioxidative properties of the samples and (v) their biocompatibility.

## 2. Materials and Methods

### 2.1. Materials

Reagents and chemicals used for the completion of this research paper involve chromium (III) chloride hexahydrate (provided by Chem-Lab NV, Zedelgem, Belgium), sodium hydroxide (provided by Sigma Aldrich, St. Louis, MO, USA), 2,2–diphenyl-1-picrylhydrazylhydrate (DPPH, provided by Sigma Aldrich, St. Louis, MO, USA) and methanol (provided by Zorka Pharma, Šabac, Serbia). The raw plant material used, chia seeds, was produced by Estyria Naturprodukte GmbH, Wollsdorf, Austria.

### 2.2. Methods

#### 2.2.1. Synthesis of Chromium Oxide Nanoparticles (CrNPs)

Chromium oxide nanoparticles were synthesised using a combination of precipitation and calcination methods. In a typical procedure, 200 mg of CrCl_3_ was dissolved in 50 mL of distilled water under continuous magnetic stirring at a temperature of 50 °C. 2 mL of 2 M aqueous solution of NaOH was added dropwise to the chromium chloride solution, which resulted in the formation of a turquoise precipitate of chromium(III) hydroxide. The resulting suspension was stirred for an additional 3 h at the same temperature to ensure complete precipitation. NaOH was used because of its proven efficiency as an effective precipitating agent, particularly for the formation of various insoluble metal hydroxides from aqueous solutions containing metal ions [27,28].

The precipitate was then isolated by centrifugation at 1250× *g* for 5 min and washed 3 times with distilled water to remove residual salts (namely, NaCl). The resulting solid chromium hydroxide was dried for 12 h under ambient temperature and pressure. Dried chromium hydroxide was ground into a fine powder using a mortar and pestle and subsequently calcined at 650 °C for 1 h (with the initial heating rate of 10.8 °C/h) in order to yield the dark-green CrNPs. The temperature of the synthesis was set to 650 °C to ensure that the entire amount of chromium hydroxide was transformed into Cr_2_O_3_ [29,30].

#### 2.2.2. Coating CrNPs with Chia Mucilage Extract

Chia mucilage was extracted following a standard hydration and separation process, utilising a method based on previous chia mucilage studies [31,32]. Firstly, 5 g of chia seeds were soaked in 200 mL of distilled water for 1.5 h at room temperature with intermittent stirring to allow the mucilage hydrogel to fully form around the seeds. The excess water was removed via a stainer, then the hydrated seeds were first blended for 5 s and filtered.

The collected mucilage solution was then subjected to centrifugation at 1250× *g* for 5 min to remove any seed fragments or insoluble particulate matter, yielding about 80 mL of white, viscous supernatant. This supernatant was frozen at −26.2 °C and subsequently lyophilized (freeze-dried) for four hours to obtain a fine, white, highly hygroscopic powder of purified chia mucilage.

Surface-functionalized chromium oxide nanoparticles were synthesised via an encapsulation method using chia mucilage extract to enhance potential biocompatibility and stability. The coating parameters were optimised by investigating three distinct mass ratios of CrNPs to chia mucilage: 1:1, 4:1, and 10:1.

For the 1:1 coating ratio, 50 mg of lyophilised chia mucilage powder was dissolved in 45 mL of distilled water under continuous magnetic stirring to yield a homogeneous solution. This solution was subsequently combined with a separately prepared aqueous suspension containing 50 mg of presynthesised chromium oxide nanoparticles dispersed in 5 mL of water.

For the 4:1 and 10:1 mass ratios, 80 mg of chromium oxide nanoparticles were combined with 20 mg of chia mucilage, and 91 mg of chromium oxide was combined with 9 mg of chia mucilage, respectively, utilising the same total volumes of distilled water as detailed for the 1:1 ratio.

Each mixture was homogenised using a high-speed homogeniser for 5 min at room temperature. The chromium oxide nanoparticle suspension was added dropwise to the chia mucilage solution during homogenization to ensure uniform dispersion and adequate surface adsorption. After homogenization, the coated nanoparticles were isolated from unbound mucilage via centrifugation at 1250× *g* for 5 min. The resulting pellet was washed twice with deionised water to remove residual unbound material and subsequently dried at room temperature and pressure [33,34].

#### 2.2.3. Characterisation Techniques

##### X-Ray Diffraction (XRD)

XRD diffractograms were recorded for the intermediate precipitate and the calcinated, non-coated CrNPs, to confirm their expected crystalline nature. For these purposes, X-ray diffraction was used, with a Philips PW 1050 diffractometer (Philips Analytical, Almelo, The Netherlands) with Cu-Kα_1,2_ radiation (Ni filter). The measurements were carried out in the 2θ range of 10° to 70°, with a scanning step width of 0.05°, and 2 s per step.

##### Fourier Transform Infrared (FTIR) Spectroscopy

FTIR spectrum of chromium oxide nanoparticles coated with chia mucilage was recorded, along with spectrum of uncoated chromium oxide nanoparticles and chia mucilage for comparison. FTIR spectra of the samples were recorded in the range of 400–4000 cm^−1^ using a Nicolet™ iS™50 FT-IR Spectrometer (Thermo Fisher Scientific Inc., Waltham, MA, USA).

##### Scanning Electron Microscope (SEM)

The scanning electron microscopy (SEM) analysis was performed on SCIOS 2 Dualbeam, Thermo Scientific, Waltham, MA, USA to characterise surface morphology, determine particle size distribution, and assess the success of the coating process for the synthesised material, to get a closer insight into the morphology of the bulk material precursor, uncoated nanoparticles, and coated nanoparticles.

##### Energy-Dispersive X-Ray Spectroscopy (EDS)

Energy-Dispersive X-ray Spectroscopy (EDS) (Oxford Instruments Aztec, High Wycombe, UK) analysis was performed jointly with SEM to verify the elemental composition of the synthesised composite material and to confirm the presence and distribution of the organic chia mucilage coating on the surface of the inorganic chromium(III) oxide nanoparticles.

##### Laser Diffraction Scattering Particle Size Analysis (LD-PSA)

The particle size analysis (PSA), performed on Malvern Mastersizer 2000 (Malvern Instruments Ltd., Malvern, Worcestershire, UK) optical bench using the laser diffraction (LD) technique (Ne-He laser source, λ  =  633 nm), was used to provide insight into the quantitative measurement of the hydrodynamic diameter of the particles in a liquid suspension (namely, isopropyl alcohol). The comparison between the physical size (as determined by SEM) and the hydrodynamic size (as determined by PSA) helps to determine the extent of swelling of the mucilage coating and the degree of solvation of the final composite material.

#### 2.2.4. Biological and Biochemical Assessment

##### *Artemia salina* Biocompatibility Assay

For testing the biocompatibility of the chromium chloride precursor, CrNPs, CrNPs-CM, and chia mucilage (CM), we used the *Artemia salina* in vivo assay. *Artemia salina* are saltwater crustaceans that have been widely used in toxicity assessment, and have been successfully used in testing the toxicity of nanoparticles as well [35]. Before the assay, *Artemia* eggs (Dajana Pet, Bohuňovice, Czech Republic) were immersed in the 30 ppm salt water for 12 h, as described in [36]. The hatched *Artemia* larvae were divided into groups and exposed to different concentrations (0.019 μg/mL to 2.5 mg/mL) of chromium precursor, CrNPs, CrNPs-CM, and CM in the wells of 96-well plates. The samples were prepared by dispersion in salt water. All experiments were performed in multiplicate, and every group contained at least 30 organisms, divided into subgroups of ~5 per well. After 24 h, the number of dead larvae was assessed by using digital microscopy DigiMicro 2.0 (Drahtlose Nachrichtentechnik, Dietzenbach, Germany) and software AMCap V9.23, and the larvae showing no movement within 30 s were considered dead. The control group was the one in which the organisms were incubated under the same conditions, but without the nanoparticles. The results were expressed as a percentage of survival compared to the non-treated, control group, with the LD50 values. LD50 is a standard quantitative measure of acute toxicity, defined as the dose of a substance required to cause death in 50% of a test population under specified conditions. It provides a comparative basis for assessing the relative toxic potency of different compounds. Although LD_50_ does not describe sublethal or long-term effects, it remains a widely used benchmark in toxicological evaluation. It is considered that toxicity in Artemia expressed by LD50 < 250 µg/mL indicates the presence of biological activity. Furthermore, values of LD50 > 20 µg/mL are considered safe for humans [36].

##### 2,2-Diphenyl-1-picerylhydrazyl Radical (DPPH•) Scavenging Assay

2,2-diphenyl-1-picrylhydrazylhydrate (DPPH•) was used as a free radical model in an assay for determining the antioxidative potential of test compounds. DPPH• molecules can accept the hydrogen atom, changing the colour from purple to yellow following this reaction. The relative amount of reduced DPPH• can be determined by measuring the decrease in absorbance intensity at 517 nm. The test was performed as described in [37], with some adaptations. The precursor, CrNPs, CrNPs-CM and CM were dispersed in water with the aid of ultrasound. DPPH• solution was prepared in methanol as a 200 µM stock. 200 µL of DPPH• solution was added to 800 µL of each sample, or in 800 µL of water for the negative control. Final concentrations were 2.5 mg/mL, 250 μg/mL and 50 μg/mL. Ascorbic acid was used as a positive control for test efficiency. Samples were incubated for 30 min, after which all of them were centrifuged at 1250× *g* for 5 min at 15 °C, and the absorbance was measured at 517 nm using a GBC Cintra UV–Vis spectrophotometer. The free radical scavenging activity was calculated by the formula:

Scavenging activity (%) = 100 × (Ac − At)/Ac

in which the Ac represents the measured absorbance of the negative control, and At represents the absorbance of the sample.

##### Ferric Cyanide (Fe^3+^) Reducing Antioxidant Power (FRAP)

To evaluate the potential of Cr, CrNPs, CrNPs-CM and CM to reduce the iron ions, we used the Ferric Cyanide (Fe^3+^) Reducing Antioxidant Power (FRAP) Assay. In this test, the reaction of the antioxidant acting via donation of electrons to Fe^3+^ oxidant, leading to reduction to Fe^2+^, is visualised by colour change from yellow to Prussian blue. Samples were processed as described in [37], including modification in using water to dilute the samples due to low methanol solubility. Final concentrations were 50 µg/mL, 250 µg/mL and 2.5 mg/mL. Reaction suspensions were centrifuged at 1250× *g* for 5 min to remove the particles from the solution. Ascorbic acid was used as a positive control, and a mixture without the test samples was used as a negative control.

##### Statistical Analysis

The antioxidative and biocompatibility assays were expressed as the average values of the acquired results, ±standard deviation. To analyse the statistical significance between the samples, Student’s *t*-test was used to compare it to the negative control values. The level of statistical significance was * *p* < 0.05.

## 3. Results and Discussion

### 3.1. Characterisation

#### 3.1.1. XRD

The XRD analysis was first performed on the sample collected as an intermediate precipitate during the synthesis process, to verify the chemical species formed in situ and confirm the proposed reaction mechanism. The established mechanism hypothesises the formation of a chromium(III) hydroxide precursor, which, upon calcination, transforms into the desired final product. The analysis was then performed on the final product obtained after the calcination.

**Figure 1:**
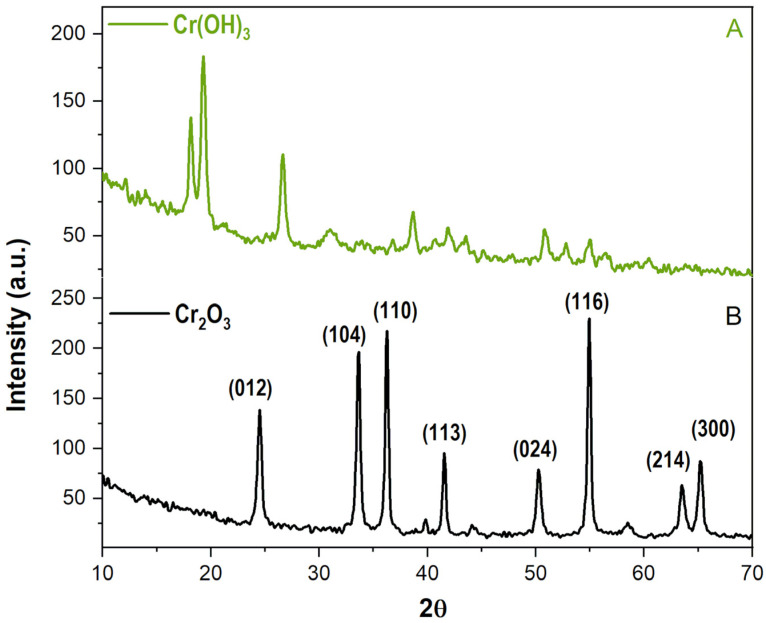
The diffractograms of (**A**) the intermediate precipitate Cr(OH)_3_ and (**B**) the calcinated sample Cr_2_O_3_.

The resulting diffractogram of the intermediate precipitate observed in Figure 1A presented a distinct pattern characterised by broad, diffuse peaks, with two of its most intense peaks positioned at approximately 18 and 19° 2θ, and another at 27° θ. A detailed comparison of this pattern to the PDF database proved a successful match with chromium (III) hydroxide (PDF number: 12-241) (Figure S1 Supplementary), thereby validating the proposed synthesis pathway and reaction kinetics.

The collected X-ray diffractogram of the final product, as seen in Figure 1B displayed a series of distinct, sharp diffraction peaks. Most intense peaks at situated at around 24.5, 33.5, 36.3, 41.5, 50, 54.5, 63.5 and 65° 2θ. Analysis and matching of these peaks with the COD databases uniquely identified the material as chromium (III) oxide (COD number: 96-901-6610, Figure S2 Supplementary), indicating complete phase transformation. The absence of characteristic peaks for any other chromium compounds in the calcined product confirms the high purity of the final material and the efficacy of the calcination process in producing a pure chromium (III) oxide phase.

The XRD analysis primarily focuses on identifying the crystalline structure and phase purity of the inorganic core material. When analysing the composite system consisting of chromium oxide nanoparticles coated with lyophilised chia mucilage, collecting XRD data specifically for the mucilage component itself becomes redundant due to the fundamental nature of the biopolymer coating. Chia mucilage is a natural polysaccharide hydrocolloid composed primarily of complex carbohydrates (sugars like xylose, arabinose, and uronic acids) and some proteins. These biopolymers possess a largely amorphous or semi-crystalline molecular structure, lacking the regular, repeating, long-range atomic order necessary to produce sharp, distinct diffraction peaks in a standard powder X-ray diffractogram.

#### 3.1.2. FTIR

**Figure 2:**
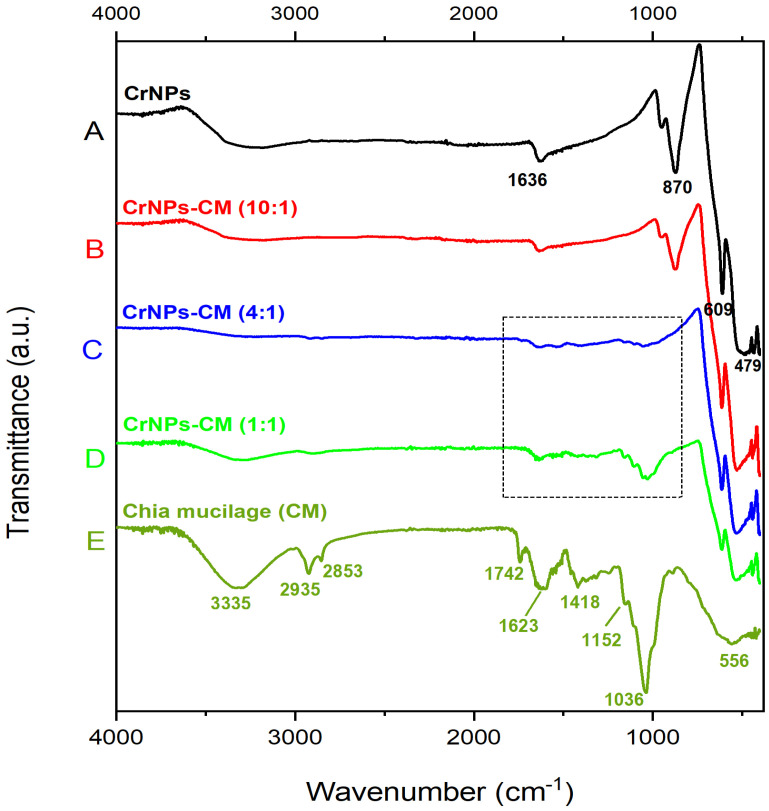
FTIR spectrum of uncoated CrNPs (**A**), coated CrNPs-CM in a 10:1 (**B**), 4:1 ratio (**C**), 1:1 (**D**) and lyophilized chia mucilage (**E**).

Figure 2 shows the FTIR spectrum of uncoated CrNPs (A), coated CrNPs-CM in a 10:1 ratio (B), 4:1 ratio (C), 1:1 ratio (D) and lyophilised chia mucilage (E). The spectrum of uncoated CrNPs at Figure 2A shows four prominent peaks: at 1636 cm^−1^, 870 cm^−1^, 609 cm^−1^, and 479 cm^−1^. The peaks at 609 and 479 cm^−1^ are the characteristic peaks for chromium oxide [38,39], and as well as the one at 870 cm^−1^, which originates from the Cr=O stretch present in the molecule [40]. Only the peak at 1636 cm^−1^ cannot be attributed to the pure chromium oxide, but is an extrinsic feature, primarily a result of environmental moisture interacting with the surface of the sample [41]. Also, the lack of other peaks intense characteristic for water or Cr(OH)_3_ intermediate, indicates that the 1636 cm^−1^ peak is present due to the atmospheric moisture. The spectrum of lyophilised chia mucilage, as seen in Figure 2E, displays a great number of peaks, which are expected for complex organic samples such as this. The broad peak observed at approximately 3335 cm^−1^ in the FTIR spectrum of chia mucilage is a characteristic band attributed to the stretching vibrations of hydroxyl (-OH) groups. The peaks at 2932 and 2823 cm^−1^ are due to C-H stretching vibrations of the alkyl groups. The peak observed at approximately 1742 cm^−1^ in the FTIR spectrum of chia mucilage is a crucial band that indicates the presence of carbonyl stretching vibrations from uronic acid residues or minor esterified components. The peaks at 1623 and 1418 cm^−1^ in the FTIR spectrum of chia mucilage are bands primarily attributed to ionised carboxylate groups. The 1152 cm^−1^ peak is a carbohydrate band connected with the C-O stretching vibrations. The peak observed at approximately 1036 cm^−1^ is a characteristic and vital absorption band for polysaccharides, primarily associated with the C-O stretching vibrations and C-O-C glycosidic ether linkages [42,43]. The region of the spectrum below 1000 cm^−1^ is a part of the “fingerprint” region and does not correspond to standard functional group vibrations like O-H or C=O stretches. Instead, the band at 556 cm^−1^ is generally attributed to complex skeletal modes of the polysaccharide backbone [44].

In the FTIR spectra of the samples coated with CM, two distinct spectral patterns can be distinguished. The first pattern shows nearly identical features to the spectrum of the uncoated CrNPs, with all characteristic bands preserved and without noticeable shifts. This spectrum was recorded for the sample with a 10:1 mass ratio (CrNPs:CM), indicating minimal presence of CM at the surface (Figure 2B). Conversely, the other two coated samples exhibit changes including the disappearance of the band at 870 cm^−1^ and the low-intensive change in the spectral line in the 1000–1700 cm^−1^ region. This region contains characteristic vibrational modes originating from CM polysaccharides. The intensity of these new bands increases with increasing CM content. Accordingly, in the 1:1 mass ratio sample (Figure 2D), the bands in this region become more pronounced, together with the appearance of peaks at approximately 2900 and 3300 cm^−1^, which are also present in the spectrum of pure CM.

The low-intensity peaks above the 610 cm^−1^ recorded in the spectrum of CrNPs-CM 4:1 (Figure 2C), can be explained by the fact that, when the nanoparticles are effectively coated by the mucilage polymer matrix, the free vibration of the atomic lattices and complex skeletal modes are physically restricted [45,46]. The strong physical interactions between the mucilage coating and the nanoparticle surface interfere with the ability of some modes to vibrate freely and absorb infrared radiation as distinct peaks. Therefore, the possible explanation for the missing peaks is that the spectral signal is effectively suppressed or masked by the physical interaction at the interface [47]. It is also worth mentioning that one chemical species can mask the peaks of other species, especially if the first species is significantly more abundant (chromium oxide is four times more abundant than chia mucilage) or has a very strong absorbance coefficient.

Results of the FTIR analysis for noncoated chromium oxide particles confirm their pure chemical structure (with a slight presence of atmospheric moisture). A series of peaks in the chia mucilage FTIR spectra clearly showcase that it is a complex organic mixture. CM is a mixture of polysaccharides (a type of carbohydrate) composed mainly of sugar units like xylose, glucose, and uronic acids, all of which contain the mentioned functional groups. Upon closer observation, the change in the spectral line between 1000 and 1700 cm^−1^ is noticed, in the same region where most of the peaks of lyophilised chia mucilage exist. This effect becomes more pronounced with increasing CM concentration, indicating more efficient coating and a higher amount of organic material on the surface of the CrNPs. In the 4:1 mass ratio sample, the presence of the organic component is only weakly observable, while in the 1:1 ratio sample, the coating agent can be clearly confirmed.

It should be noted that these ratios represent the initial mass ratios used during synthesis rather than the final composition of the coated particles. As described in the synthesis procedure, after the coating step, the particles were separated by centrifugation and washed twice to remove excess unbound biopolymer. As a result, only the fraction of the biopolymer that interacts with the particles through adsorption and other non-covalent interactions remains associated with the surface. These interactions are strong enough to withstand the washing process.

The applicability of FTIR spectroscopy for stability evaluation of natural and bio-derived materials has also been demonstrated through its successful use in monitoring structural integrity during storage, as recently reported for amaranth extracts [48]. The stability of the obtained samples was evaluated by comparing FTIR spectra of freshly prepared and two-month-aged samples of both uncoated CrNPs and coated CrNPs (4:1) stored at room temperature (Figures S3 and S4 Supplementary). No detectable spectral differences were observed between the initial and aged powders. The characteristic bands associated with Cr–O vibrations and low-intensity peaks that originate from surface polysaccharide functional groups remained unchanged with respect to position and intensity, indicating that no appreciable chemical modifications occurred during storage.

The preservation of spectral features confirms that neither oxidation of the chromium oxide phase nor degradation of the CM coating took place over the tested period. Such stability is particularly advantageous for practical applications, as it implies that both uncoated and coated nanoparticles retain their functional properties upon prolonged storage without requiring controlled environmental conditions.

#### 3.1.3. SEM

After the XRD confirmed that the synthesis process yielded the desired chemical composition, SEM analysis was performed on the samples of CrNPs-CM, with different mass ratios of CrNPs to CM (1:1, 4:1, or 10:1), as well as on the chromium chloride used as precursor in the synthesis and the non-coated CrNPs just for comparison.

**Figure 3:**
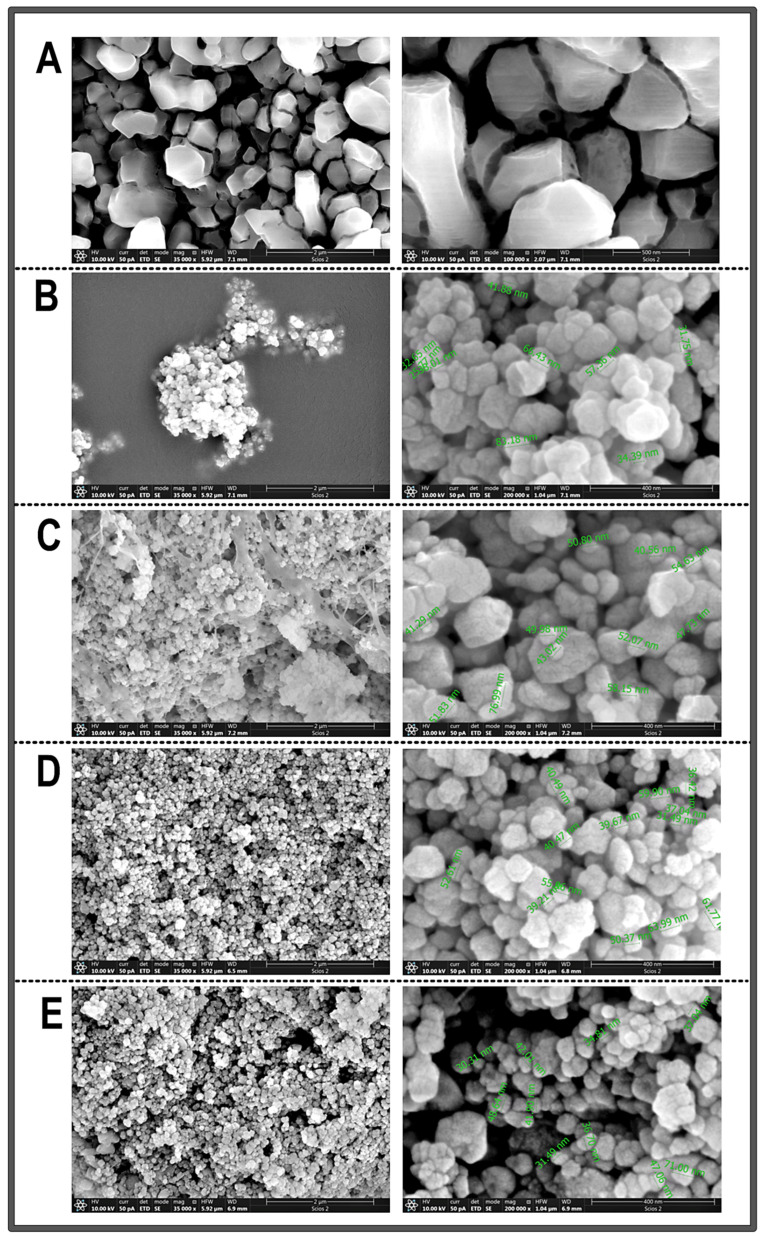
Representative SEM images of samples obtained under different magnifications (**A**) bulk material CrCl_3_ (bars 2 µm and 500 nm), (**B**) non-coated CrNPs (bars 2 µm and 400 nm), (**C**) CrNPs-CM 1:1 (bars 2 µm and 400 nm), (**D**) CrNPs-CM 4:1 (bars 2 µm and 400 nm), (**E**) CrNPs-CM 10:1 (bars 2 µm and 400 nm).

SEM micrographs in Figure 3A confirmed that the precursor was in a bulk form, which was important for the later comparison with the synthesised samples. Micrographs in Figure 3B revealed that non-coated chromium oxide samples consist of nano-scale particles, which, however, appear to be highly agglomerated and concentrated in one place. Particles appear to be predominantly spherical or quasi-spherical in shape. Measured particles exhibit a size distribution with diameters roughly ranging from 25 to 83 nm. Micrographs in Figure 3C significantly differ from the micrographs of non-coated particles. The addition of chia seed mucilage in a 1:1 mass ratio to the CrNPs provided the strap-like network structures around the chromium oxide nanoparticles. Measured particles exhibited a narrower size distribution than the non-coated particles, with diameters roughly ranging from 40 to 76 nanometers. However, a substantial amount of submicron agglomeration was still observed. Micrographs in Figure 3D showed that particles synthesised in a 4:1 ratio show high uniform distribution and constant shape and morphology, without the appearance of network structures, as was the case with the sample CrNPs-CM 1:1. Measured particles exhibited an even narrower size distribution than the previous two samples, with diameters ranging approximately from 40 to 65 nm. The particles are, as in previous micrographs, dominantly spherical in shape. Micrographs in Figure 3E portray slightly more agglomerated particles, with less uniform distribution. Measured particles exhibited a similar size distribution to the previous examples.

All these results indicate that the sample CrNPs-CM with a mass ratio of 4:1 yielded particles with the most consistent morphology, narrowest size distribution, and least agglomeration, which can be attributed to the optimal concentration of the chia mucilage stabilising agent. At the 4:1 ratio, the concentration of the mucilage was likely sufficient to provide adequate steric hindrance and electrostatic repulsion between the individual nanoparticles during the coating process. The polymeric chains of the mucilage effectively adsorbed onto the surface of the chromium oxide nanoparticles, creating a protective layer that counteracted the natural tendency of nanoparticles to aggregate due to their high surface area and surface free energy. In contrast, other ratios presented suboptimal conditions (the appearance of strap-like network structures and higher agglomeration). This controlled nanoscale dimension is critical for applications where high surface area and quantum effects are desired, and it confirms the efficacy of the chosen synthesis method for producing materials within the specified range [49].

#### 3.1.4. EDS

**Figure 4:**
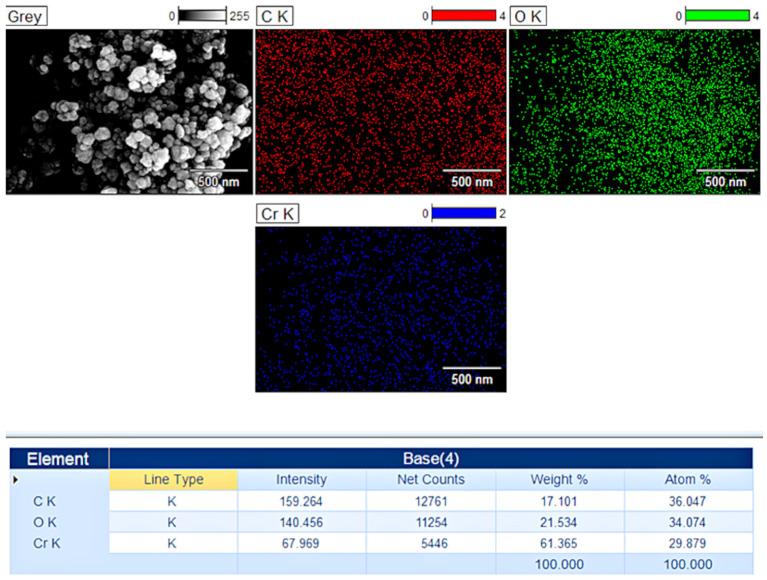
Mapping images and elemental analysis for CrNPs-CM with a 4:1 ratio.

The analysis of the CrNPs-CM prepared at an optimised 4:1 mass ratio, as shown in Figure 4, demonstrated a proportional presence of chromium, oxygen, and carbon. The most significant finding from the EDS analysis, in the context of the coating objective, was the distinct presence of elements characteristic of the organic mucilage layer for the 4:1 ratio. The spectrum showed noticeable amounts of carbon. The lyophilised mucilage, being a polysaccharide, is rich in carbon and oxygen; the increase in the overall elemental ratio relative to the pure confirms the formation of a core–shell structure. EDS elemental mapping provided further spatial confirmation. The mapping images for the sample CrNPs-CM 4:1 ratio illustrated that the chromium and oxygen signals were highly localised to the nanoparticle cores, while the carbon signal exhibited a uniform distribution that completely enveloped these inorganic cores. This spatial overlap indicates that the mucilage forms a consistent encapsulating layer rather than merely co-existing as separate, aggregated organic debris.

In light of the data provided by the SEM and EDS analyses, further characterisation was performed only on CrNPs-CM 4:1, as it proved the most optimal ratio.

#### 3.1.5. LD-PSA

**Figure 5:**
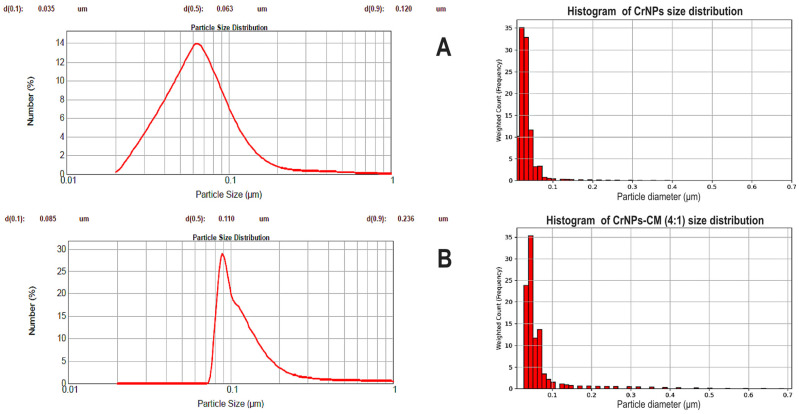
Particle size distribution by number % of non-coated CrNPs (**A**), and CrNPs-CM 4:1 sample (**B**), and their corresponding histograms.

The distribution in Figure 5A shows that the non-coated CrNPs mean diameter is concentrated around 0.063 µm, which is consistent with the SEM results. As seen in Figure 5B, CrNPs-CM 4:1 mean diameter is concentrated around 0.110 µm, which is larger than the diameter of the non-coated particles, and thereby it is also an indication of the successful coating of the particles.

When discussing the results of the LD-PSA, it is evident that coated particles are larger in diameter, which is the indication that the coating of the particles was successfully performed. The slightly larger diameter than expected (compared to the SEM results) can be explained by the fact that the particles were analysed while being dispersed in a fluid, so consequently, the value measured by the instrument is actually their hydrodynamic radius, i.e., their size as the particle move through a liquid, including any layers of solvent that move with it, contributing to the diameter [50].

These measurements also revealed a narrow particle size distribution for the synthesised material. This narrow distribution, which was even more pronounced in the coated particles, is a robust indicator of the system’s high colloidal stability and sample homogeneity [51].

### 3.2. Biological and Biochemical Assessment

The samples examined by *Artemia salina* biocompatibility assay and DPPH• test were the precursor used in the synthesis of CrNPs, which is CrCl_3_, non-coated chromium oxide nanoparticles (CrNPs), lyophilised chia mucilage (CM), and chia mucilage-coated chromium oxide nanoparticles in a 4:1 mass ratio (CrNPs-CM 4:1).

#### 3.2.1. *Artemia Salina* Biocompatibility Assay

The LD50 results were as follows: 0.22 for CrCl_3_, 23.29 for CrNPs, and 1.65 for CM. The CrNPs-CM toxicity was too low, and thus the LD50 for this species could not be calculated. The LD50 values were calculated as provided in the literature [35].

**Figure 6:**
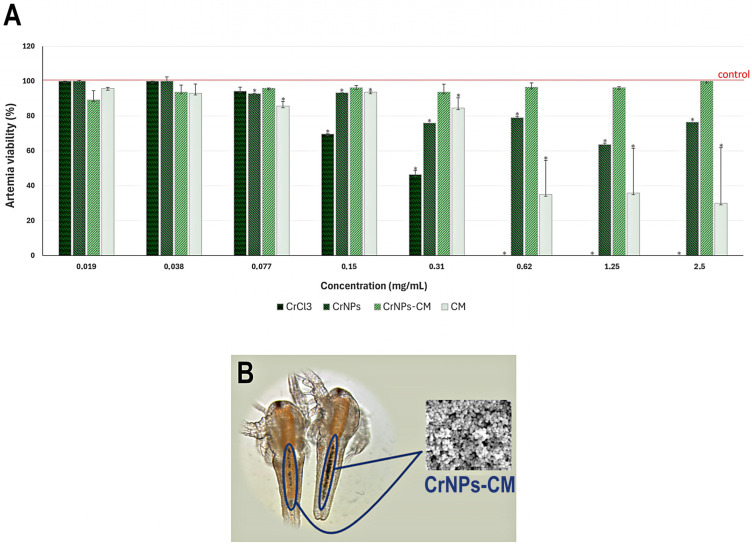
(**A**) Survival of *Artemia* larvae treated with 0.019 μg/mL to 2.5 mg/mL of CrCl_3_, CrNPs, CrNPs-CM 4:1 and CM, expressed as a percentage of survival relative to the negative control group. Statistical significance threshold was *p* < 0.05 and was represented by *. (**B**) 24 h old Artemia salina nauplii treated with 0.31 mg/mL of CrNPs-CM; the organism has ingested a significant amount of particles, leading to their accumulation in its digestive tract, as visible on the optical microscope. However, there were no observable behavioural changes, no morphological or other visually noticeable toxic effects.

The results of the biocompatibility testing on Artemia salina featured significant variation in the survival between the groups treated with different samples, as seen in Figure 6A. There were no surviving organisms at more than 0.31 mg/mL in the case of precursor, the CrCl_3_. Furthermore, significant morphological changes were observed even at a lower tested concentration of CrCl_3_. The CrNPs-treated organisms exhibited significantly higher survival rates, with the lowest survival at 1.25 mg/mL being around 60%. However, there was almost no change, compared to the negative control, in the groups treated with CrNPs-CM 4:1. The survival was above 90% at all tested concentrations. The CM itself did not cause significant mortality at lower concentrations, but this was followed by a drop in survival to below 40% at concentrations higher than 0.31 mg/mL.

Figure 6B presents 24 h-old Artemia salina nauplii exposed to 0.31 mg/mL CrNPs-CM. Optical microscopy reveals pronounced particle ingestion, with clear accumulation within the digestive tract. Despite this uptake, no behavioural alterations, morphological abnormalities, or other visually detectable toxic effects were observed. The results suggest that the CrNPs-CM 4:1 have excellent biocompatibility, which is crucial when considering their future applications. While the precursor sample proved completely lethal at concentrations above 0.62 mg/mL, the non-coated CrNPs had a significantly higher survival rate, with the CrNPs-CM 4:1 sample having almost no lethality at either concentration. Chia gel became solidified at higher concentration, making survival hard for the immersed Artemia larvae, which is the probable cause of the sudden drop in survival at concentrations above 0.31 mg/mL, and not the chemical toxicity itself.

It is also worth mentioning that Artemia salina biocompatibility assay has been compared to the MTT assay on human cell lines, giving similar results [52]. The Artemia salina lethality assay even offers an important advantage over the MTT assay by providing an integrated, whole-organism assessment of toxicity that captures systemic and developmental effects, whereas MTT is limited to measuring metabolic activity at the cellular level in vitro [53].

#### 3.2.2. DPPH and FRAP Assays

**Figure 7:**
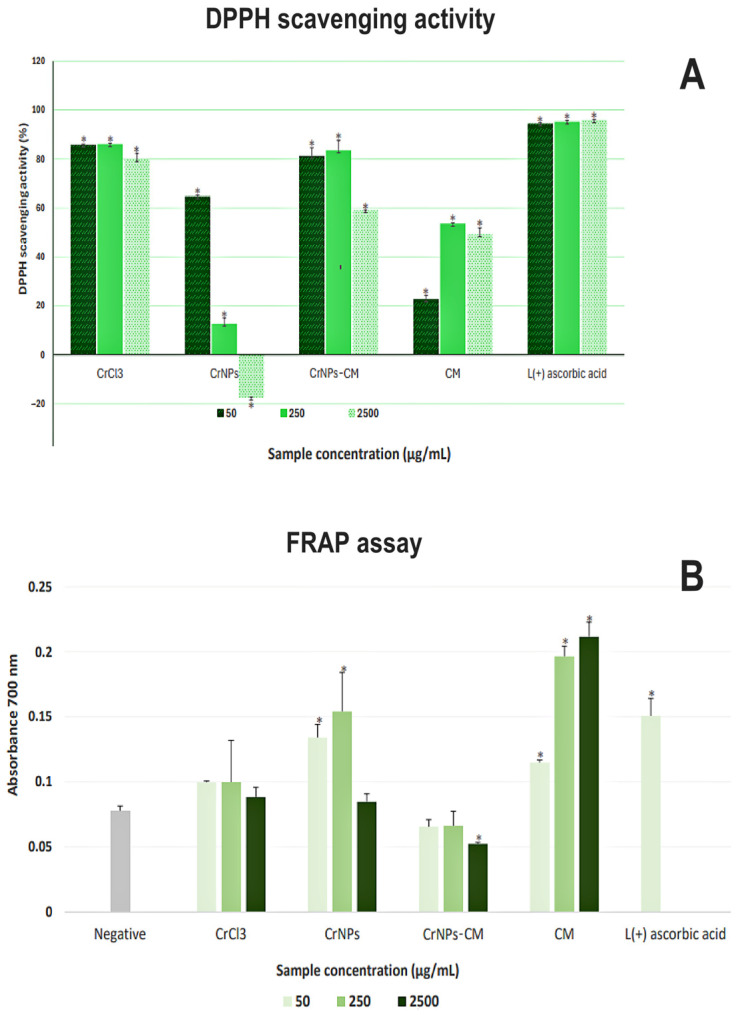
Results of the assessment of antioxidant activity (**A**) The DPPH• scavenging activity (%) of Cr, CrNPs, CrNPs-CM and CM was calculated in comparison to the absorbance of the control DPPH• solution, which was used as an 0% value. Statistical significance compared to the control solution was * *p* < 0.05. Ascorbic acid was used as a positive control. (**B**) FRAP assay, expressed as the absorbance values at λ = 700 nm of the supernatant solutions after reaction of 50 µg/mL, 250 µg/mL and 2.5 mg/mL of Cr, CrNPs, CrNPs-CM and CM with potassium ferrocyanide, with the increase in A700 indicating the level of reduction of Fe^3+^ to Fe^2+^. Statistical significance compared to the negative control was set to * *p* < 0.05. Ascorbic acid was used as a positive control.

The results of the DPPH• reduction assay have shown high antioxidative activity of the precursor (above 80% of scavenging) at all concentrations, without significant concentration dependency, as witnessed in Figure 7A. On the other hand, CrNPs had a lesser effect, with negative concentration dependency. The CrNPs-CM 4:1 exhibited high activity (more than 80% of free radical scavenging) at 250 μg/mL and 50 μg/mL, but at the highest concentration of 2.5 mg/mL, it was lower. The CM sample did not reach 60% of free radical scavenging at all concentrations, but had positive concentration dependency. As the CM sample itself did not have overly significant antioxidative activity, one can postulate that the relatively high activity of sample CrNPs-CM 4:1 does not come exclusively from coating. In the study of [54], the CrNPs nanoparticles were synthesised by using pomegranate husk as a capping and reducing agent, resulting in particles that also had high free radical scavenging activity comparable to those synthesised in this research, but featuring positive concentration dependency. It is possible that smaller-sized nanoparticles, remaining in the solution after the centrifugation, could add to the absorbance.

The results of the FRAP assay, as shown in Figure 7B, revealed a distinct trend in the antioxidative activity of the samples compared to DPPH•. Only CM exhibited a noticeable concentration-dependent activity. CrCl_3_ did not show statistically significant activity compared to the negative control. CrNPs showed low activity at lower concentrations, and the CrNPs-CM exhibited no activity, with the absorbance of the highest concentration being even lower than that of the negative control. The activity of 250 µg/mL and 2.5 mg/mL of ascorbic acid was higher than the instrument threshold.

This discordance in results is very scientifically significant, as it indicates that CrNPs and CrNPs-CM 4:1 antioxidant mechanism is primarily driven by radical quenching rather than metal-reducing power [55]. Furthermore, these results suggest that both CrNPs and CrNPs-CM predominantly exert their antioxidant activity through a hydrogen atom transfer (HAT) mechanism. Since the FRAP assay is based exclusively on an electron transfer (ET) process, it cannot capture HAT-mediated chain-breaking antioxidant effects or the specific radical-scavenging capacity of the tested materials [56].

Taken together with the DPPH• results (Figure 7A), the presented findings indicate a synergistic antioxidant effect arising from the specific surface association between CrNPs and CM. The DPPH scavenging capacity of CrNPs-CM exceeded those of both components individually, suggesting that the polysaccharide coating enhances radical quenching efficiency beyond simple additive contributions. This enhancement may arise from specific surface localisation and favourable orientation of hydroxyl- and carbonyl-containing groups at the nanoparticle interface. Moreover, the markedly improved biocompatibility of CM-coated CrNPs compared to bare CrNPs underscores the functional significance of this surface engineering approach, supporting the potential of CrNPs-CM as safer and more effective antioxidant agents.

Additionally, FTIR spectroscopy confirmed that polysaccharides and uronic acids are the dominant components of CM, whose hydroxyl and carbonyl groups can participate in radical scavenging. When bound to CrNPs, these groups are likely involved in coordination or hydrogen bonding to the nanoparticle surface, limiting their reducing capacity but enabling efficient quenching of free radicals.

## 4. Conclusions

This study establishes a simple, innovative approach for preparing chromium (III) oxide nanoparticles tailored for biomedical and pharmaceutical use. Chromium oxide is known for its chemical stability, redox activity, and durability, yet its biomedical potential has remained largely unexplored due to challenges in dispersion and biocompatibility.

By coating Cr_2_O_3_ nanoparticles with chia seed mucilage extract, this work resolves those limitations and creates a platform that preserves the intrinsic advantages of chromium while ensuring safe interaction with biological systems. Physicochemical analyses (XRD, FTIR, SEM, EDS, and LD-PSA) confirmed the successful synthesis of Cr_2_O_3_ nanoparticles and their coating by chia mucilage. The 4:1 nanoparticle to chia mucilage mass ratio produced well-defined spherical particles with minimal aggregation and a consistent size of about 50 nm. Biological testing further highlighted the benefits of this system. The coated nanoparticles (CrNPs-CM 4:1) showed strong antioxidant activity, based on a radical quenching mechanism, and excellent biocompatibility, demonstrating that the natural polysaccharide coating not only stabilises chromium oxide but also enhances its suitability for biological use.

In this work, the successful transformation of chromium(III) oxide nanoparticles from a chemically promising yet biologically limited material into a safe, functional nanoplatform is reported. These findings open new possibilities for using chromium-based nanomaterials in targeted biomedical applications, such as controlled delivery systems, antioxidant therapies, and future multifunctional nanodevices.

## Data Availability

The original contributions presented in this study are included in the article/Supplementary Material. Further inquiries can be directed to the corresponding author.

## Supplementary Materials

The following supporting information can be downloaded at: https://www.mdpi.com/article/10.3390/pharmaceutics18010049/s1, Figure S1: Phase identification of chromium(III) hydroxide using the PDF database (PDF No. 12-241). Figure S2: Matching of the calcinated sample Cr_2_O_3_ with the COD databases uniquely identified the material as chromium(III) oxide (COD number: 96-901-6610). Figure S3: FTIR spectra of freshly prepared and two-month-aged uncoated CrNPs sample stored at room temperature. Figure S4: FTIR spectra of freshly prepared and two-month-aged coated CrNPs (4:1) stored at room temperature.
