# Supplementary material for: Synthesis, Characterisation, and Biological Assessment of Chromium Oxide Nanoparticles Coated with Chia Seed Mucilage Extract
Source: Pharmaceutics. 2025 Dec 30;18(1):49. doi: 10.3390/pharmaceutics18010049 (PMC12845127; doi:10.3390/pharmaceutics18010049)

Synthesis, characterisation, and biological assessment of chromium oxide nanoparticles coated with chia seed mucilage extract

Sara Lukač [1], Nina Tomić[1], Zoran Stojanović[1], Vladimir Rajić[2], Nenad Filipović[1], Maja Jović[1], Magdalena Stevanović[1]*

[1] Group for Biomedical Engineering and Nanobiotechnology, Institute of Technical Sciences of SASA, Kneza Mihaila 35/IV, 11000 Belgrade, Serbia
[2] Department of Atomic Physics, Vinča Institute of Nuclear Sciences, University of Belgrade, Mike Petrovića Alasa 12-14, 11000 Belgrade, Serbia
* Correspondence: magdalena.stevanovic@itn.sanu.ac.rs

Supplementary material

Figure S1 Phase identification of chromium (III) hydroxide using the PDF database (PDF No. 12-241).

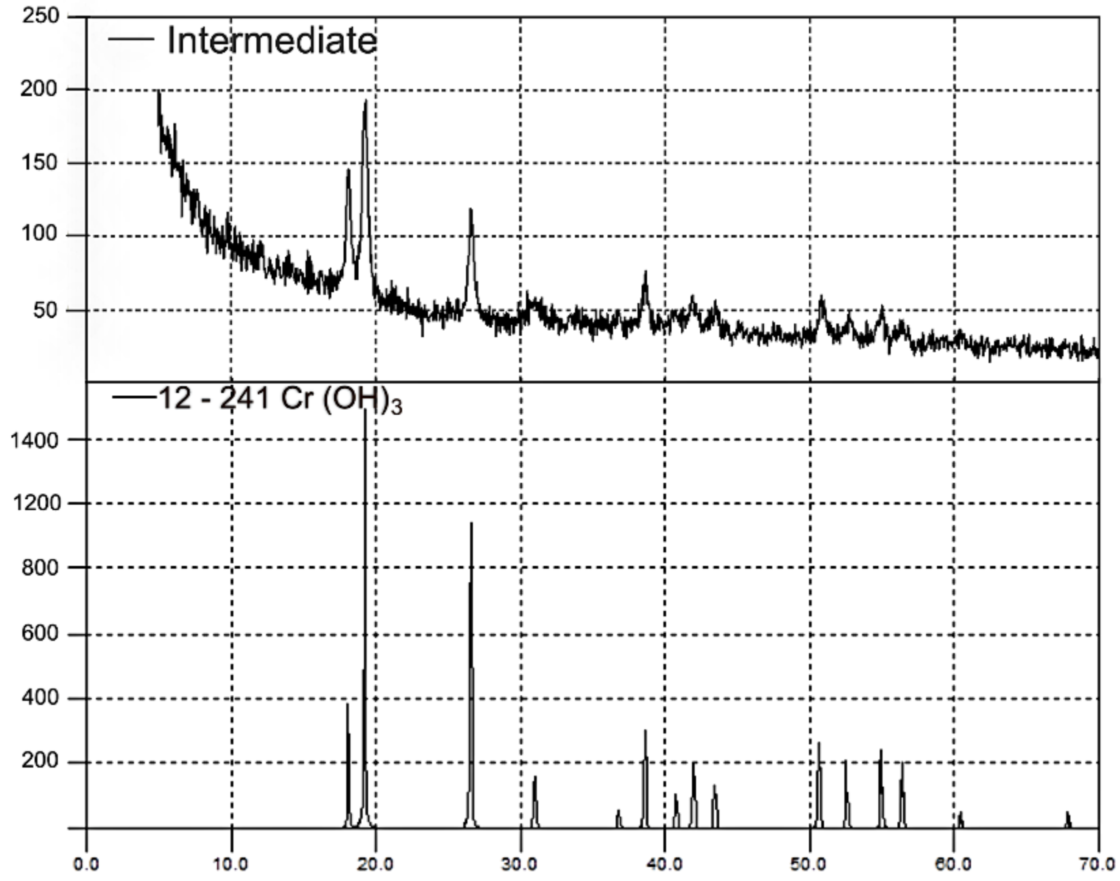

Figure S2 Matching of the calcinated sample Cr_2O_3 with the COD databases uniquely identified the material as chromium (III) oxide (COD number: 96-901-6610)

Match! Phase Analysis Report

Institute of Technical Sciences, Serbian Academy of Sciences and Arts

Sample: MAJACR3H

Sample Data

File name	CR3H.DAT
File path	C:/Rendgen/
Data collected	2025 15:29:22
Data range	10.000° - 80.000°
Original data range	10.000° - 80.000°
Number of points	1401
Step size	0.050
Rietveld refinement converged	No
Alpha2 subtracted	No
Background subtr.	No
Data smoothed	No
Radiation	X-rays
Wavelength	1.541874 Å

Analysis Results

Index	*Amount (%)*	*Name*	*Formula sum*
A	100.0	Eskolaite	Cr2 O3

Amounts calculated by RIR (Reference Intensity Ratio) method

Element	*Amount (weight %)*
Cr	68.4%
O	*31.6%(*)*
**LE (sum)*	*31.6%*

Diffraction Pattern Graphics

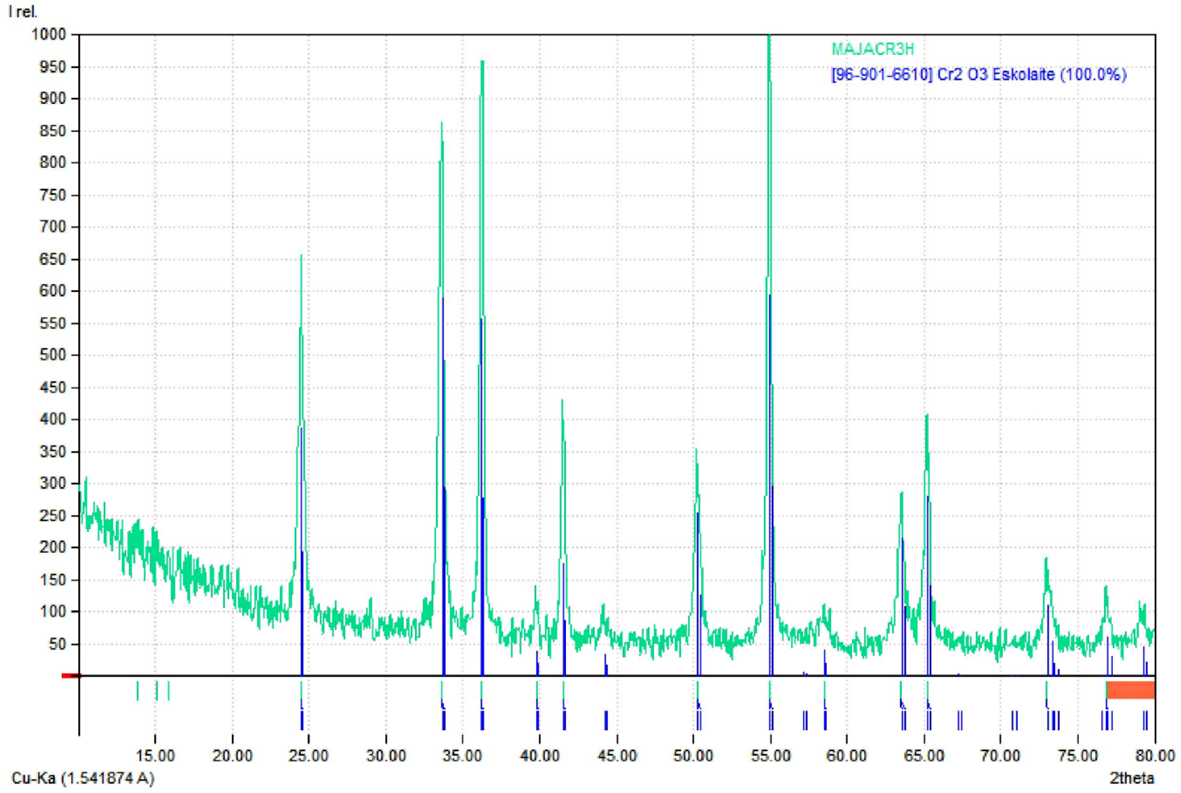

Match! Copyright © 2003-2025 CRYSTAL IMPACT, Bonn, Germany

Figure S3 FTIR spectra of freshly prepared and two-month-aged uncoated CrNPs sample stored at room temperature

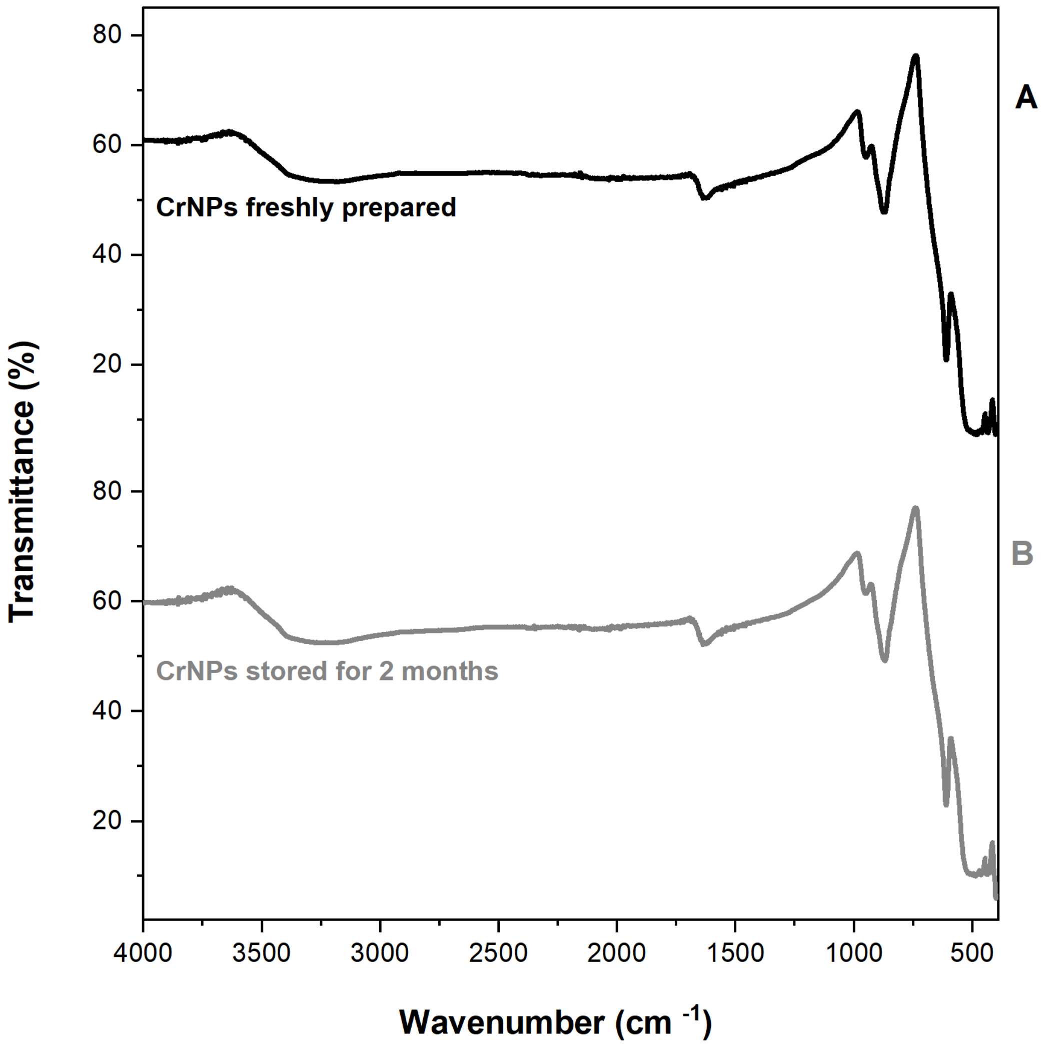

Figure S4 FTIR spectra of freshly prepared and two-month-aged coated CrNPs (4:1) stored at room temperature

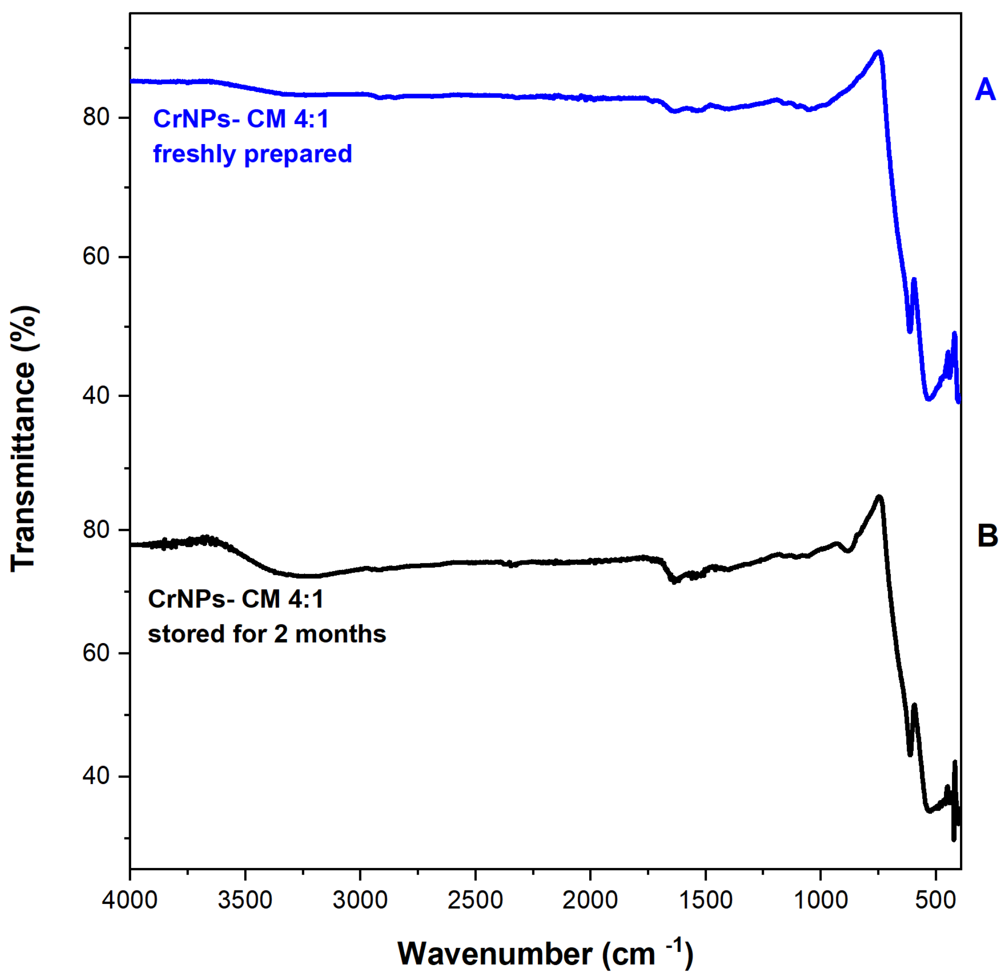

Supplement: Supplementary file 1 [file pharmaceutics-18-00049-s001.zip › pharmaceutics-4061621-supplementary.pdf]
